# RELATIONSHIPS BETWEEN PERCEIVED HOUSING, HEALTH, AND QUALITY OF LIFE: DATA FROM AGES 65–70 IN SWEDEN

**DOI:** 10.1093/geroni/igad104.0735

**Published:** 2023-12-21

**Authors:** Erik Eriksson, Maya Kylén, Steven Schmidt

**Affiliations:** Lund University, Lund, Skane Lan, Sweden; Lund University, Lund, Skane Lan, Sweden; Lund University, Lund, Skane Lan, Sweden

## Abstract

Theoretical and empirical advances have supported the notion of the importance of the perceived environment, and in particular the housing environment, as we age. For instance, previous research has found significant associations between aspects of perceived housing, such as control beliefs and meaningful aspects of home, and health and wellbeing. These associations have been found for both younger older, and older older, adults. However, much of previous research has been cross-sectional, in particular for younger older adults, leaving the question whether this association remains over time as we age. By using data from a Swedish longitudinal study (N=371, 57 % women) we analyzed associations between Meaning of Home (MOH) and two external subscales of Housing-Related Control Beliefs (HCQ), to a 6-year change in 31 self-reported health symptoms and health-related quality of life, measured by SF-12, using linear regression and modelling for demographic and housing characteristics. Significant results were found between both housing-related control beliefs and meaning of home, to changes in metabolism symptoms (MOH, R2 = 0.07, F(9, 259) = 3.258, p = .001; HCQ, R2 = 0.072, F(9, 281) = 3.494, p < .000 and R2 = 0.066, F(9, 280) = 3.263, p = .001). However, for all other health symptom groups and for health-related quality of life, no significant associations were found. Our results show that while perceived aspects of housing are associated with health at a given point in time, they may not be predictive of future changes in health status.

